# A Pilot Study on Electrical Impedance Tomography During CPAP Trial in Patients With Severe Acute Respiratory Syndrome Coronavirus 2 Pneumonia: The Bright Side of Non-invasive Ventilation

**DOI:** 10.3389/fphys.2021.728243

**Published:** 2021-09-09

**Authors:** Michela Rauseo, Lucia Mirabella, Donato Laforgia, Angela Lamanna, Paolo Vetuschi, Elisa Soriano, Daniele Ugliola, Elena Casiello, Livio Tullo, Gilda Cinnella

**Affiliations:** Department of Anesthesia and Intensive Care, University of Foggia, Foggia, Italy

**Keywords:** SARS-CoV-2, electrical impedance tomography, awake prone positioning, CPAP, non-invasive ventilation

## Abstract

**Background:** Different severe acute respiratory syndrome coronavirus 2 (SARS-CoV-2) pneumonia phenotypes were described that match with different lung compliance and level of oxygenation, thus requiring a personalized ventilator setting. The burden of so many patients and the lack of intensive care unit (ICU) beds often force physicians to choose non-invasive ventilation (NIV) as the first approach, even if no consent has still been reached to discriminate whether it is safer to choose straightforward intubation, paralysis, and protective ventilation. Under such conditions, electrical impedance tomography (EIT), a non-invasive bedside tool to monitor lung ventilation and perfusion defects, could be useful to assess the response of patients to NIV and choose rapidly the right ventilatory strategy.

**Objective:** The rationale behind this study is that derecruitment is a more efficient measure of positive end expiratory pressure (PEEP)-dependency of patients than recruitment. We hypothesized that patients who derecruit significantly when PEEP is reduced are the ones that do not need early intubation while small end-expiratory lung volume (ΔEELV) variations after a single step of PEEP de-escalation could be predictive of NIV failure.

**Materials and Methods:** Consecutive patients admitted to ICU with confirmed SARS-CoV-2 pneumonia ventilated in NIV were enrolled. Exclusion criteria were former intubation or NIV lasting > 72 h. A trial of continuos positive airway pressure (CPAP) 12 was applied in every patient for at least 15 min, followed by the second period of CPAP 6, either in the supine or prone position. Besides standard monitoring, ventilation of patients was assessed by EIT, and end-expiratory lung impedance (ΔEELI) (%) was calculated as the difference in EELI between CPAP_12_ and CPAP_6_. Tidal volume (Vt), Ve, respiratory rate (RR), and FiO_2_ were recorded, and ABGs were measured. Data were analyzed offline using the dedicated software. The decision to intubate or continue NIV was in charge of treating physicians, independently from study results. Outcomes of patients in terms of intubation rate and ICU mortality were recorded.

**Results:** We enrolled 10 male patients, with a mean age of 67 years. Six patients (60%) were successfully treated by NIV until ICU discharge (Group S), and four patients failed NIV and were intubated and switched to MV (Group F). All these patients died in ICU. During the supine CPAP decremental trial, all patients experienced an increase in RR and Ve. ΔEELI was < 40% in Group F and > 50% in Group S. In the prone trial, ΔEELI was > 50% in all patients, while RR decreased in Group S and remained unchanged in Group F.

**Conclusion:** ΔEELI < 40% after a single PEEP de-escalation step in supine position seems to be a good predictor of poor recruitment and CPAP failure.

## Introduction

At present time, more than 1 year since the severe acute respiratory syndrome coronavirus 2 (SARS-CoV-2) pandemic broke out, its physiopathology and clinical course are much better understood. However, to speak with Sir W. Churchill, “the best is not good enough,” since many key points of this threatening disease are still far from being fully elucidated, notably as regards therapeutic options, while the number of cases that require respiratory support is far from being subsided. Hence, clinicians all over the world still have to face the impossibility to guarantee tracheal intubation for everyone (Grasselli et al., 2020), and non-invasive respiratory support becomes sometimes an obliged choice. Presently, an increasing amount of studies are reporting successful treatment of SARS-CoV-2 pneumonia in non-intubated patients, ventilated with moderate to high level of positive airways pressure (PEEP/CPAP) (8–12 cmH_2_O) and cycles of awake proning (Bamford et al., 2020; Whang et al., 2021). Accurate selection of patients, appropriate PEEP/CPAP setting, and correct timing for switching to invasive mechanical ventilation in non-responders to the non-invasive approach are thus of paramount importance and still remain among the most debated topics in the intensive care unit (ICU) environment and more (Lee et al., 2021; Tseng et al., 2021).

Hypoxemia severity and oxygenation response to PEEP/CPAP application are weak indexes of lung recruitability (Dantzker, 1982; Chen et al., 2020), and this is even more evident in SARS-CoV-2 pneumonia, since patients with a same degree of severity of arterial desaturation may present with different clinical phenotypes, ranging from normal respiratory rate (RR) to marked dyspnea and near normal to decreased respiratory system compliance (Crs), where compliance is a measure of the lung expandability. It refers to the ability of the lung to stretch and expand (Gattinoni et al., 2020a,b,c). In fact, the most challenging patients are the ones that under non-invasive ventilation keep normal PaCO_2_, RR, and adequate minute ventilation (12–14 l/min) (He et al., 2019), with preserved lung mechanics, in whom PaO_2_/FiO_2_ do not respond to the application of increasing PEEP levels. These patients are at high risk for developing self-inflicted lung injury (PSILI) (Brochard et al., 2017; Grieco et al., 2019; Yoshida et al., 2020) and require intubation as early as possible. Assessing if a patient is recruiter or not can thus guide the decision to keep non-invasive ventilation or intubate. A recruiter can be defined as a patient who responds to PEEP in terms of better oxygenation, hemodynamic stability, improvement of respiratory compliance, and lung mechanics. However, lung recruitability is uneasy to measure at the bedside and even more difficult in non-intubated patients. Recently, Chen et al. (2020) proposed a single-breath bedside method to measure lung recruitment by using the so-called compliance of the recruited lung: the ratio between the loss in end-expiratory lung volume (ΔEELV) after a reduction of PEEP, and driving pressure (DP) itself. However, this R/I index can be used only in sedated and mechanically ventilated patients. We hypothesized that the same physiological principle could be applied to patients under non-invasive ventilation with PEEP/CPAP, to screen the ones that are PEEP-dependent: after a drop in PEEP/CPAP, a reduction in EELV, and thus an increase in ΔEELV, should indicate that lung maintains a good elasticity and is able to deform. These lungs are affected by the pressure change and respond to the PEEP, so the clinician should insist with CPAP maximized at 12 cmH_2_O (He et al., 2019). In contrast, a stable EELV, and thus a small ΔEELV, with no changes in other parameters such as RR or Ve, would indicate that patients have an airway opening pressure (AOP) > 12 cmH_2_O (Chen et al., 2018) and thus PEEP must be increased over 12 cmH_2_O to reach alveoli, or that lungs are already stiff and fibrotic. These patients should be better rapidly switched to invasive mechanical ventilation. Thus, a delay in understanding this process, in a patient with spontaneous breathing, may worsen PSILI or may delay intubation and reduce the chances for survival. Under this hypothesis, ΔEELV could be a predictor of CPAP failure and the need for switching to invasive mechanical ventilation.

Electrical impedance tomography (EIT) is a non-invasive and easy to use bedside tool that dynamically shows regional tidal volume (Vt) distribution. EIT has been demonstrated a valid instrument to assess regional ventilation and PEEP-induced recruitment in many experimental and clinical studies (Cinnella et al., 2015; Sosio et al., 2019). However, to our knowledge, no data are available on the use of EIT to monitor lung recruitability under non-invasive ventilation in patients with COVID-19.

Thus, we launched the present observational pilot study in patients admitted in ICU with SARS-CoV-2 pneumonia treated by non-invasive ventilation with PEEP/CPAP and cycles of the prone position. This study was aimed at (a) estimating PEEP-dependency at the bedside by using EIT to measure ΔEELV by end-expiratory lung impedance variations (ΔEELI) after a drop in PEEP; (b) evaluating the relationship between PEEP-dependency and outcome of patients, in terms of intubation and survival rate; (c) assessing if EIT may be useful to identify responders to the prone position, by continuously monitoring the ventilation redistribution following changes of the position.

## Materials and Methods

### Patients

After Ethics Committee approval and written informed consent, all consecutive patients with COVID-19 admitted to our academic hospital ICU from March 2021 to April 2021 were enrolled. Inclusion criteria were as follows: admission from the emergency room within 24 h from symptoms onset, non-invasive mechanical ventilation for clinical decision, age ≥ 18 years, and confirmed SARS-CoV-2 infection from a respiratory tract sample by PCR-based tests. Exclusion criteria were as follows: prior admission to the ward, prior intubation, hemodynamic instability, defined as systolic blood pressure (BP) < 90 mmHg or mean BP < 60 mmHg, contraindication to EIT use (presence of pacemaker), impossibility to correctly place EIT belt, and refusal to participate in this study.

Patients underwent standard monitoring: ECG, heart rate (HR), RR, FiO_2_, SaO_2_, and urinary output. The radial artery was cannulated, and the catheter connected to the pressure transducer of the MostCare monitor (MostCareup, Vigon, Italy) and/or the IntelliVue Philips X3 monitor (Philips Medizin Systeme Böblingen GmbH, Böblingen Germany).

Intravascular pressure measurements were adjusted to zero at atmospheric pressure and leveled to the mid-axillary line. Analysis of arterial blood gases was performed (GEM Premier 4000, Werfen, United Kingdom).

All patients were ventilated using a Respironics V60 Ventilator (Philips N.V., Netherlands) in CPAP mode and connected to a full-face mask (Respironics FitLife, Philips) as the interface. Vt, RR, and minute ventilation (Ve) were measured from the ventilator.

Electrical impedance tomography is a non-invasive imaging technique that gives you a special view of inside the lungs. In a cross-sectional projection, the distribution of the tidal volume in the thorax is shown. The derived image shows ventilated and non-ventilated areas of the lungs and their changes as a function of time.

The EIT (PulmoVista 500, Draeger Medical GmbH, Germany) was applied as follows: a rubber belt containing 16 electrodes was placed around the chest between the fourth and fifth intercostal space and connected to the EIT monitor (Draeger/GoeMFII EIT Evaluation Kit 2, Draeger Medical GmbH). The correct position and signal quality were assessed on the monitor screen as described (Cinnella et al., 2015). At every study step, the EIT images were divided into four quadrants, to obtain two ventral and two dorsal regions of interest (ROIs), as already described (Cinnella et al., 2015; Sosio et al., 2019). Real-time impedance curves represent ventilation over time. Changes in the overall cross-section are reflected by the global impedance curve. This curve strongly correlates with the volume curve of the ventilator and with the applied/inhaled total volume. The regional impedance changes (i.e., tidal variations) serve to compare different lung regions. The numerical values indicate the volume distribution, which together adds up to 100% of the global value unless the overall window size (ROI setting) is changed.

### Data Analysis

**FIGURE 1:**
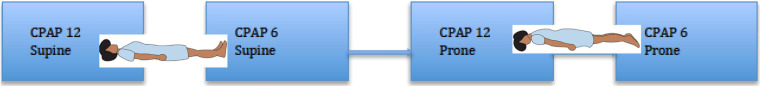
Study steps protocol.

The operator was always the same. We recorded EELI according to a validated method (Sosio et al., 2019). Later, the patient was connected to a V60 Philips Respirator, and the recordings were taken in the four study steps (Figure 1).

The Dräger (SW EITdiag V1.6 (Draeger Lübeck, Germany), is a dedicated software tool for advanced PC-based analysis of EIT data files that have been previously recorded with PulmoVista 500 or other devices applying the technique of electrical impedance tomography. EITdiag reconstructs EIT images and uses various previously published approaches for data interpretation with respect to regional and temporal inhomogeneity of the lung function (Lowhagen et al., 2010).

An offline analysis was performed with the EITdiag software on impedance data to calculate global lung impedance and ΔEELI. The end-inspiratory trend view is used to compare two different tidal images and their regional tidal volume distribution. It helps to identify inhomogeneities, recruitment, derecruitment, overdistension, and the redistribution of Vt when changing the PEEP setting on the ventilator.

Recorded data included demographics [age, gender, and body mass index (BMI)], comorbidities, previous pharmacological treatments, disease chronology [time from onset of symptoms and from hospital admission to initiation of respiratory support, and ICU length of stay (LOS)], symptoms at ICU admission, vital signs [temperature, mean arterial pressure (MAP), and heart rate], laboratory parameters (blood test, coagulation, and biochemical), non-respiratory sequential organ failure assessment (non-respiratory SOFA) and APACHE II scores, and outcome in ICU and hospital.

### Interventions

A physician not involved in this study was responsible for the care of patients. All patients were awake and mildly sedated with dexmedetomidine 0.3–1.2 mcg/kg/h. A standardized protocol for hemodynamic management was applied to ensure fluid volume optimization, as already described (Cinnella et al., 2015).

**FIGURE 2:**
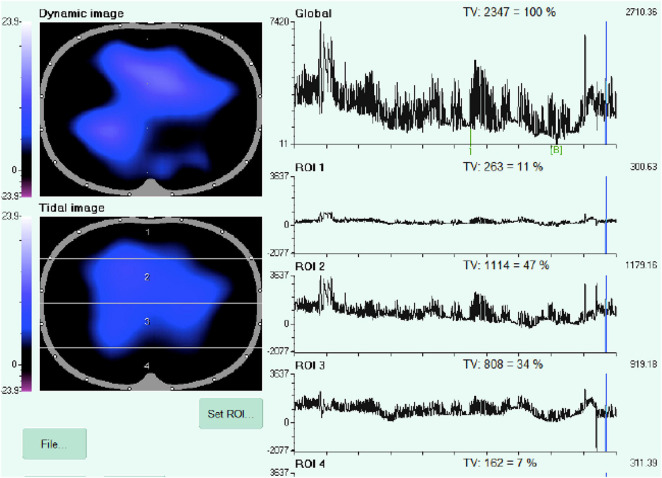
EIT ventilation dynamic distribution image from one CPAP decremental trial. The screenshot shows the distribution of the tidal volume in a cross sectional full screen view of the patient thorax in the caudal-cranial direction.

This study was performed in four steps as follows (Figures 1, 2): (a) in the supine position, CPAP was set at 12 cmH_2_O, and the first series of the measurement was performed (T1s); (b) CPAP was then decreased to 6 cmH_2_0, and the series of the second measurement was recorded (T2s); (c) patients were then turned to the prone position, CPAP was again set to 12 cmH_2_O, and the third series of measurements was performed (T1p); (d) CPAP was decreased to 6 cmH_2_O (T2s), and the last measurement was performed. Every step lasted 15 min. The whole procedure lasted 1 h plus the time necessary for turning patients to the prone position. After the final steps, the physician in charge decided whether the patients can be kept in the prone position or not and set CPAP according to her/his clinical judgment. The decision to switch to intubation and invasive mechanical ventilation was taken by treating physicians independently from study results.

During every step, Vt, RR, and minute ventilation (Ve) were recorded together with data from the EIT as already described (Cinnella et al., 2015). BP, HR, SaO_2_, and a baseline ABG were collected, together with blood screen of the day, as per ward policy.

### Statistical Analysis

Data are expressed as percentage, mean ± SD, since they are normally distributed (Shapiro–Wilk test, *p* > 0.05). We used the one-way ANOVA to assess differences between CPAP_12_ and CPAP_6_ and differences between supine and prone positions. A *p* value < 0.05 was considered significant. Statistical analysis was performed using Statistica 10.0 (TIBCO software Inc., Palo Alto, CA, United States; Statsoft Italia srl 2011; available at: www.statsoft.com).

## Results

In this study, 10 men patients were included, with a mean age of 67 (range 51–81) years, a weight of 85 ± 20 kg, and a BMI of 20 ± 15.

The mean time lapse from the appearance of symptoms to ICU admission was within a range of 7–10 days.

Six patients were successfully discharged from the ICU to ward after 12 ± 2 days, without being intubated (Table 1). The remaining four patients failed the non-invasive ventilation (NIV) trial, were intubated, and mechanically ventilated within 48 h post trial. All these patients died in ICU (mean ICU stay 14 ± 3.5 days, range 14–20 days.

**TABLE 1:** Demographics data and outcome.

**Patient**	**Sex**	**Age **(y-o)****	**BMI **(Kg/m^2^)****	**Comorbidities**	**Outcome**
1	M	80	33	HTN	ICU discharge
					Day 12th
2	M	57	28	None	ICU discharge
					Day 7th
3	M	79	32	HTN	Dead
					Day 10th post ETT
4	M	77	30	HTN	ICU discharge
					Day 12th
5	M	80	26	HTN, CKI	Dead
					Day 4th post ETT
6	M	68	33	HTN	Dead
					Day 21st
					(24 h post ETT)
7	M	60	29	HTN, DM II	Dead
					Day 32nd
8	M	51	28	None	ICU discharge
					Day 6th
9	M	55	29	None	ICU discharge
					Day 9th
10	M	60	26	HTN	ICU discharge
					Day 5th

*BMI, body mass index; ETT, endotracheal tube; HTN, hypertension; CKI, chronic kidney injury; DM II, Diabetes Mellitus Type II.*

In the whole group, baseline PaO_2_/FiO_2_ was 180 ± 20 and remained unchanged during the study (NS). RR and Ve increased when going from CPAP 12 to CPAP 6, both in supine (RR 18 ± 2 in T1s to 28 ± 2 bpm in T2s; Ve 8.3 ± 1.2 to 12.5 ± 3.2 L × min^–1^) and prone positions (RR 17 ± 2 in T1p to 26 ± 3 bpm in T2p; Ve 8.5 ± 0.6 to 11.6 ± 2.7 L × min^–1^; *p* < 0.05), while Vt remained stable in every patient (Table 2).

**TABLE 2:** Respiratory parameters during CPAP trials, in both positions.

	**Supine**		**Prone**		***P* value**
	**CPAP 12 cmH_2_O**	**CPAP 6 cmH_2_O**	**CPAP 12 cmH_2_O**	**CPAP 6 cmH_2_O**	
Vt (ml)	450 ± 100	430 ± 150	500 ± 80	460 ± 90	*P* = 0.2
RR (breaths per minute)	18 ± 2	28 ± 2^*^	17 ± 2	26 ± 3^*^	*P* < 0.05
Ve (l/min)	8.3 ± 1.2	12.5 ± 3.2^*^	8.5 ± 0.6	11.6 ± 2.7^*^	*P* < 0.05
PaO_2_/FiO_2_	180 ± 20	170 ± 20	220 ± 10	190 ± 10	NS
ΔEELI Global (%) NIV failure	28 ± 4		63 ± 9 #		*P* < 0.01
ΔEELI Global (%) NIV success	58 ± 6		62 ± 8 #		*P* = 0.05

^*^CPAP_12_ vs. CPAP_6_;

*^#^ Supine vs. Prone position. NS, not significant.*

**FIGURE 3:**
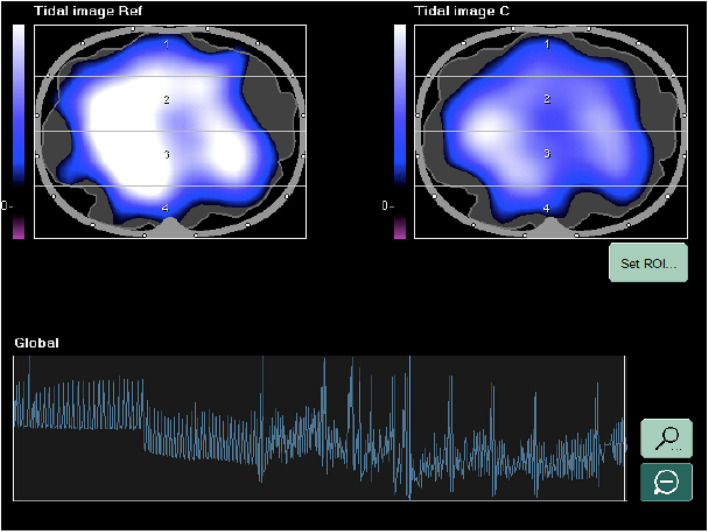
Analysis from one CPAP decremental trial and change in ventilation distribution in a patient who failed NIV and died (the huge variability of the signal in the final step of the trial highlights the fatigue).

**FIGURE 4:**
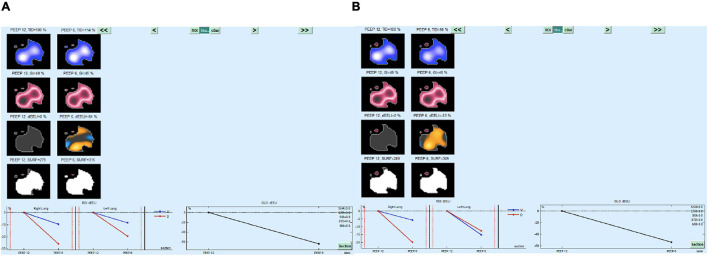
EITdiag MatLAb Analysis of the decremental CPAP trial in a patient who survived, **(A)** supine, **(B)** prone. Note the ΔEELI% > 50 in both body positions.

**FIGURE 5:**
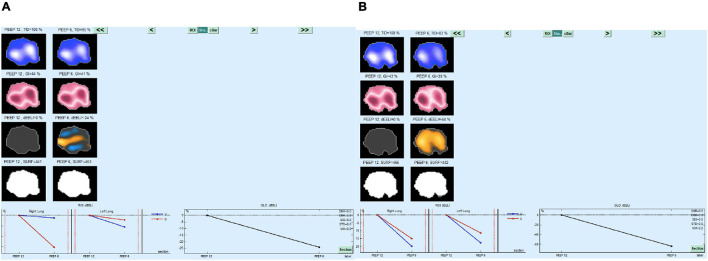
EITdiag MatLAb Analysis of the decremental CPAP trial in a patient who died, **(A)** supine, **(B)** prone. Note the ΔEELI% < 40 in supine postion and > 50 in prone postion.

*A posteriori* analysis of survivors vs. non-survivors showed that ΔEELI (Table 2) was > 50% in all survivors and remained stable in both supine and prone positions (58 ± 6% and 62 ± 8%, respectively, *p* = 0.05). In non-survivors, ΔEELI was 28 ± 4%, with small or no changes in the supine trial, and increased up to 63 ± 9% (<0.01) during the prone trial. Ve increased on the last phase of the trial (Figure 3). In Figures 4, 5, EITdiag MatLab analysis from two representative patients (one survivor and one non-survivor) is shown.

The four patients intubated were ventilated with 6 ml/kg/PBW and PEEP of 12 ± 2 cmH_2_O. Measurement of respiratory mechanics showed a plateau pressure (Pplat) < 30 cmH_2_O in all patients, with mean static compliance (Cs) of 40 ± 4 ml/cmH_2_O; patients #3 and 7 had an AOP of 13 ± 2 cmH_2_O, and patients # 5 and 6 were not recruiters (R/I < 0.5).

## Discussion

The main findings of the present pilot study are that patients with SARS-CoV-2 pneumonia under non-invasive ventilation may have two distinct behaviors to a CPAP decremental trial: (a) ΔEELI > 50% either in the supine or prone position, indicating lung recruitability, associated with NIV success; (b) ΔEELI < 40% in the supine position, indicating non-recruiter lungs (phenotype L) or AOP > 12 cmH_2_O, associated with NIV failure; (c) in patients who failed NIV, an increased ΔEELI% exclusively during proning may be due to a better V/Q matching.

The novelty of our study is that we performed a decremental CPAP trial in awake SARS-CoV-2 patients and used EIT, a non-invasive, bedside tool that does not require specific competencies in respiratory mechanic assessment, to assess the changes in lung volume. Thus, we applied in our patients the same physiological principle used in intubated and mechanically ventilated patients to discriminate between recruiters and non-recruiters (Perier et al., 2020; van der Zee et al., 2020; Kotani and Shono, 2021; Shono et al., 2021).

Before discussing our results, a short excursus on NIV state-of-the-art in SARS-CoV-2 pneumonia is required. In fact, the time course of findings from trials focused on non-invasive respiratory support reflects the difficulties that everybody, everywhere, had to face in front of a new syndrome of such a sprawling aspect as SARS-CoV-2. Retrospectively, one can bitterly meditate on the ATS/ERS guidelines (Rochwerg et al., 2017), on the use of NIV in *de novo* acute respiratory failure and critical viral illness pandemic statement: “*we are unable to offer a recommendation*,” just before the SARS-CoV-2 pandemic explosion. Later, the panel added: *“*…*we consider prior recommendations against the use of NIV for pandemic as unsupportable.*” Nonetheless, the worldwide fight against SARS-CoV-2 started from ATS/ERS first statement, so that the earliest international guidelines on COVID 19 (Whang et al., 2021) did not recommend NIV in such patients. However, it became soon evident that the situation had gone out of control and 1,000 patients had to be treated non-invasively because the alternate choice was not respiratory support at all. Paradigmatic of this struggle is the amendments made by National COVID-19 Clinical Evidence Taskforce (NCCET) (Whang et al., 2021) that deleted the following statement from its revised guidelines “*in patients with hypoxemia associated with COVID-19, do not routinely use NIV*” (Whang et al., 2021). Since then, a number of studies dealt with the COVID-19 outcome prediction (Tseng et al., 2021), early vs. late intubation (Lee et al., 2021), and criteria for NIV and prediction of NIV failure (Bellani et al., 2017; He et al., 2019), so that at present, the debate is still going and no definite recommendations are available, while patients are still largely ventilated with NIV, mainly in non-ICU departments and often by non-ICU trained physicians. An effort to provide clear and simple means to early discriminate patients that require intubation is thus needed, perhaps more due to ethical reasons than purely speculative ones.

Severe acute respiratory syndrome coronavirus 2 pneumonia may present with two phenotypes which identification is pivotal to apply the right mechanical ventilation strategy (Gattinoni et al., 2020a): type L, characterized by low elastance, low V/Q ratio, lung weight, and recruitability, and type H, defined by high elastance, right to left shunt, lung weight, and recruitability. Recruitability is defined as the possibility to open up collapsed areas of the lung by positive pressure. The “classical” lung-protective strategy (Gattinoni et al., 2006, 2020a,2020b,2020c), based on limiting Vt and plateau pressure is not required for type L but should be actively implemented in type H. However, it is not easy to distinguish between the two types because the lung weight cannot be measured clinically, and the method to evaluate recruitability is unclear, while a method to measure V/Q matching at the bedside has not been established. Under such conditions, EIT was proposed not only to evaluate recruitability but also to assess regional ventilation homogeneity, thus allowing to determine the optimum PEEP at the time of measurement. As a result, this approach could be of help to set ventilation to attenuate regional dynamic strain and inhomogeneity of transpulmonary pressure. Morais et al. (2021) presented three cases of mechanically ventilated patients with COVID-19 with acute hypoxemic respiratory failure (AHRF) and ARDS that had similar levels of oxygenation but variable respiratory system compliance. In this case series, different characteristics of the regional ventilation profile were evidenced by EIT that was thus helpful in understanding the etiology of hypoxemia at the bedside. Tomasino et al. (2020) used EIT to identify the characteristics of COVID-19 pneumonia and to decide whether to use high PEEP or prone positioning.

In this study, PEEP de-escalation affected most of the respiratory parameters in all patients, disclosing two behaviors that matched with a clinically significant difference in the outcome of patients. Interestingly, the two behaviors were evident in the supine position and not in the prone position: the four patients that failed NIV had only slight variations in terms of ΔEELI when kept in the supine position, probably because in this position, lung stiffness, alveolar collapse, and V/Q mismatch play a major role than in prone position (Cornejo et al., 2013; Yoshida et al., 2013; Scaravilli et al., 2015; Aguirre-Bermeo et al., 2018; Telias et al., 2020). In fact, this hypothesis was confirmed by respiratory mechanic data obtained after intubation in these patients showing that two of them were not recruiters (R/I < 0.5), and the remaining two had an AOP > 12 cmH_2_O, signs of their need for mechanical ventilation and higher PEEP levels. These four patients had already signs of fatigue on their admission to the ICU, and it could be argued that NIV would probably have not been indicated for them from the beginning. However, since their PaO_2_/FiO_2_ was acceptable when compared with other patients, they underwent an NIV trial. In contrast, the six patients in whom NIV was successful (all discharged from ICU) had a significant increase in ΔEELI when PEEP was reduced, both in supine and prone positions, and in our opinion, this can be explained still by soft lungs and high recruitability, since the end-expiratory trend view or ΔEELI-trend view is used to monitor regional changes of ΔEELI. ΔEELI is strongly correlated with the changes in ΔEELV. The ΔEELI trend is useful to assess the changes in lung volume, for example, after changing the PEEP and after recruitment maneuvers for the reopening of dorsal atelectases and for the detection of derecruitment of individual lung areas.

Paradoxically, it could be argued that the prone position could mask NIV failure precisely because of its favorable effects. In fact, the physiological rationale behind prone positioning in typical ARDS is to reduce ventilation/perfusion mismatching, hypoxemia, and shunting (Cornejo et al., 2013; Scaravilli et al., 2015; Aguirre-Bermeo et al., 2018). When a patient is in the prone position, the pleural pressure gradient between dependent and non-dependent lung regions decreases as a result of gravitational effects and matching of conformational shape of the lung to the chest cavity. This generates more homogenous lung aeration and strain distribution, thus enhancing recruitment of dorsal lung units (Telias et al., 2020), while the regional distribution of pulmonary blood flow is not altered, with perfusion predominating toward the dorsal lung due to non-gravitational factors. In patients with spontaneous breathing, respiratory physiology under prone position is the same plus the effect of diaphragm contraction, since its muscular mass is mainly *posterior*, when in the prone position, it exerts on lungs a more uniform distribution of stress (Yoshida et al., 2013). Lung regional hyperinflation may thus be reduced (Telias et al., 2020). Therefore, the prone position allows an improvement in ventilatory homogeneity with a relatively constant perfusion pattern, and a subsequent reduction in shunting is observed together with an increase in EELV. In fact, none of our patients had a severe respiratory failure (they would not have been in NIV), so that all responded to proning with the expected EELV increase, but the physiological and clinical meaning of these data is different in the two groups. The benefit of prone position in terms of V/Q matching did probably overcome the role played by OAP or lung stiffness in those patients who were not recruitable, and this could explain why all did have a ΔEELI > 50% when switched from 12 to 6 cmH_2_O in pronation.

In contrast, in the supine position, the damages of lung inflammation and edema are more evident. Due to the increased weight of the lung, alveolar collapse may predominantly occur in the dependent lung regions, and the resulting arterial hypoxemia is worsened by diaphragmatic contractions that cause gas displacement from non-dependent to dependent lung areas, the so-called pendelluft phenomenon (Yoshida et al., 2013). Moreover, strong inspiratory effort causes large negative pressure in the thorax and increased transpulmonary pressure that can cause or aggravate lung injury, generating the so-called PSILI, whose pathological changes are irreversible and worsen the prognosis (Brochard et al., 2017; Grieco et al., 2019; Yoshida et al., 2020). Under such conditions, a lung that is PEEP-dependent, i.e., recruitable, will lose volume when PEEP is decreased while a lung stiff or with AOP will remain quite unaffected.

## Conclusion

Although our results need to be confirmed by larger data set and further RCTs should be conducted to evaluate whether the use of the EIT could, in fact, help to detect different phenotypes and clustering patients able to tolerate NIV, our data seem to suggest that a PEEP de-escalation trial in the supine position can be useful to discriminate lung recruitability in patients with SARS-CoV-2 under NIV.

## Data Availability Statement

The original contributions presented in the study are included in the article/supplementary material, further inquiries can be directed to the corresponding author/s.

## Ethics Statement

The studies involving human participants were reviewed and approved by Comitato Etico Azienda Ospedaliero Universitaria Policlinico Riuniti di Foggia. The patients/participants provided their written informed consent to participate in this study.

## Author Contributions

MR and GC conceived the presented idea. MR wrote the first draft. DL performed the measurements. AL and LM verified the analytical methods. PV, ES, DU, EC, and LT encouraged MR to investigate the use of the EIT in patients with COVID-19 and supervised the findings of this study. GC contributed to the final manuscript. All authors discussed the results.

## Conflict of Interest

The authors declare that the research was conducted in the absence of any commercial or financial relationships that could be construed as a potential conflict of interest.

## Publisher’s Note

All claims expressed in this article are solely those of the authors and do not necessarily represent those of their affiliated organizations, or those of the publisher, the editors and the reviewers. Any product that may be evaluated in this article, or claim that may be made by its manufacturer, is not guaranteed or endorsed by the publisher.

## Abbreviations

ΔEELI: Variation of End Expiratory Lung Impedence
ΔEELV: variation of End expiratory Lung Volume
GI: Global Impedence
ROI: Region of Interest
NIV: Non Invasive ventilation
MV: Mechanical Ventilation
RR: Respiratory Rate
Ve: Minute ventilation
Vt: Tidal Volume
PSILI: Patient Self Inflicted Lung Injury
R/I: Recruitment to Inflaction Ratio
AOP: Airway Opening Pressure
DP: Driving Pressure
BP: Blood Pressure
HR: Heart rate
ABG: Aterial Blood Gas Analysis
Crs: Respiratory system compliance
Cs: Static Compliance
V/Q: Ventilation/Perfusion ratio.
